# Genomic landscape of the *OsTPP7* gene in its haplotype diversity and association with anaerobic germination tolerance in rice

**DOI:** 10.3389/fpls.2023.1225445

**Published:** 2023-07-25

**Authors:** Kyaw Myo Aung, Win Htet Oo, Thant Zin Maung, Myeong-Hyeon Min, Aueangporn Somsri, Jungrye Nam, Kyu-Won Kim, Bhagwat Nawade, Chang-Yong Lee, Sang-Ho Chu, Yong-Jin Park

**Affiliations:** ^1^ Department of Plant Resources, College of Industrial Sciences, Kongju National University, Yesan, Republic of Korea; ^2^ Center for Crop Breeding on Omics and Artificial Intelligence, Kongju National University, Yesan, Republic of Korea; ^3^ Department of Industrial and Systems Engineering, College of Engineering, Kongju National University, Cheonan, Republic of Korea

**Keywords:** rice, *trehalose-6-phosphate phosphatase 7*, anaerobic germination, genetics, haplotype diversity, haplotype-based breeding

## Abstract

Early season flooding is a major constraint in direct-seeded rice, as rice genotypes vary in their coleoptile length during anoxia. *Trehalose-6-phosphate phosphatase 7* (*OsTPP7*, *Os09g0369400*) has been identified as the genetic determinant for anaerobic germination (AG) and coleoptile elongation during flooding. We evaluated the coleoptile length of a diverse rice panel under normal and flooded conditions and investigated the Korean rice collection of 475 accessions to understand its genetic variation, population genetics, evolutionary relationships, and haplotypes in the *OsTPP7* gene. Most accessions displayed enhanced flooded coleoptile lengths, with the temperate *japonica* ecotype exhibiting the highest average values for normal and flooded conditions. Positive Tajima’s D values in *indica*, admixture, and tropical *japonica* ecotypes suggested balancing selection or population expansion. Haplotype analysis revealed 18 haplotypes, with three in cultivated accessions, 13 in the wild type, and two in both. Hap_1 was found mostly in *japonica*, while Hap-2 and Hap_3 were more prevalent in *indica* accessions. Further phenotypic performance of major haplotypes showed significant differences in flooded coleoptile length, flooding tolerance index, and shoot length between Hap_1 and Hap_2/3. These findings could be valuable for future selective rice breeding and the development of efficient haplotype-based breeding strategies for improving flood tolerance.

## 1 Introduction

Rice (*Oryza sativa* L.) is a staple food crop predominantly cultivated and consumed in Asia and Africa and ranks as the world’s number one food crop (Anuonye et al., 2016). It provides nearly 50% of the daily caloric intake for consumers (Paul, 2020). Direct-seeded rice (DSR) is a popular cultivation method due to its low labor requirements and minimal water and energy consumption. Thus, it is an economical and environmentally friendly choice (Kumar and Ladha, 2011; Zhang et al., 2015). Despite the risk of low germination in DSR caused by soil flooding (Senapati et al., 2019), its cost-effectiveness and convenience have made it a widespread practice globally (Yang et al., 2019). However, modern rice varieties do not germinate well when submerged in water, due to the lack of coleoptile elongation and the negative impact of long periods of oxygen deprivation on root and shoot development (Magneschi and Perata, 2009). Excessive or prolonged flooding can therefore result in partial or complete submergence of seedlings, adversely affecting the germination and survival of rice seedlings (Ismail et al., 2012).

Among different abiotic stresses, flooding is one of the major constraints for rice production, especially in rainfed lowland areas, and it threatens global food security (Dar et al., 2017). Climate change has exacerbated this problem, as modern rice varieties are vulnerable to flooding (Panda and Barik, 2021). Approximately 22 million hectares of South and South-East Asia are unfavorably submerged each year, negatively impacting the livelihoods of over 100 million people (Singh et al., 2016). Around 15 million hectares of rainfed lowland areas are specifically impacted by short-term flash flooding leading to substantial production loss (Singh et al., 2017). Over 35% of the world’s rice acreage is flood prone, and much is in regions of Asia and Africa, where food insecurity is prevalent (Bailey-Serres et al., 2012; Dwivedi et al., 2016). Germination under flooding is known as “anaerobic germination” (AG), where seeds can germinate without oxygen or undergo hypoxia or anoxia. This results in poor or no germination, seedling death, and poor crop establishment (Septiningsih and Mackill, 2018). Thus AG tolerance is critical for developing direct-seeded rice, enabling robust seedling establishment through rapid and sustained coleoptile elongation in the flooded condition (Tnani et al., 2021). QTL mapping studies have identified coleoptile elongation as an indicator trait of the tolerance phenotype, with tolerant genotypes exhibiting faster coleoptile elongation under submergence (Ismail et al., 2009; Narsai et al., 2015). Conversely, sensitive genotypes are susceptible to slower coleoptile growth (Miro and Ismail, 2013). Therefore, coleoptile elongation is a key criterion for selecting AG-tolerant rice varieties.

Several research groups have identified QTLs and their stability for AG tolerance in rice (Baltazar et al., 2014; Hsu and Tung, 2015; Kim and Reinke, 2018; Ghosal et al., 2019; Yang et al., 2019; Jeong et al., 2020). One of the earliest reports was by Angaji et al., who found the QTL AG1 (anaerobic germination 1) on the *japonica* landrace, Khao Hlan On (Angaji et al., 2010). Later studies identified that the QTL AG1 is encoded by *trehalose-6-phosphate phosphatase 7* (*OsTPP7*), which enhances rice’s AG tolerance (Kretzschmar et al., 2015). Another prominent QTL for AG tolerance, AG2 (qAG7.1), located on chromosome 7, was mapped using a population derived from IR42 and the indica variety Ma-Zhan Red (Septiningsih et al., 2013). Recently, it was narrowed down from 7 Mb to less than 0.7 Mb, revealing the presence of 27 genes within this region. However, the specific candidate gene conferring AG tolerance remains to be identified (Tnani et al., 2021). While several AG tolerance loci have been identified, only one QTL (AG1) located at the long arm of chromosome 9 has been fine-mapped, cloned, and functionally validated as *OsTPP7* (Kretzschmar et al., 2015). *OsTPP7* plays a crucial role in the metabolism of trehalose-6-phosphate (T6P), a key molecule that acts as an energy sensor, regulating resource allocation for anabolism or catabolism based on sucrose availability (Kretzschmar et al., 2015; Yu et al., 2021). By modulating the balance between T6P and sucrose, *OsTPP7* influences the flux of carbohydrates from source tissues to sink tissues, thereby controlling starch mobilization through the activity of α-amylases (Paul, 2008; Yu et al., 2015; Gómez-Álvarez and Pucciariello, 2022). This fine-tuning of carbohydrate distribution by *OsTPP7* facilitates sugar transport to the embryo and the germinal sheath, leading to successful germination, vigorous early seedling growth and influencing the regulation of coleoptile length (Magneschi and Perata, 2009; Kretzschmar et al., 2015; Yu et al., 2021). Under anaerobic stress, *OsTPP7* increased the turnover of T6P, enhancing starch mobilization and driving the growth kinetics of the germinating embryo and the coleoptile elongation, which ultimately enhanced AG tolerance (Kretzschmar et al., 2015). Later, this gene was used in molecular breeding and introduced into popular high-yielding rice varieties, including IR64, to increase anaerobic germination tolerance (Toledo et al., 2015; Kim et al., 2019; Alam et al., 2020).

With the availability of NGS data, utilization of single nucleotide polymorphisms (SNPs) and insertions/deletions (InDels) has become increasingly important in population genetics, evolutionary analysis, association studies, and molecular breeding in rice (Guo et al., 2014; Kim et al., 2016; Kim et al., 2022). The primary quantitative trait of AG tolerance is associated with two genes, AG1 and AG2. A better understanding of the roles of these genes could help address the problem of flooding for AG in DSR. Researchers and breeders have utilized various strategies to identify useful genetic variations that arise through evolutionary changes in rice. Exploration of a germplasm collection is essential to understand the genetic history and diversity of particular traits, as well as to identifying valuable haplotypes that can be utilized in breeding programs (Khush, 1997; Abbai et al., 2019; Zhang et al., 2021).

Although the AG1 gene is known to have a significant role in rice breeding, there is still much to learn about its genetic variation, population genetics, and evolutionary relationships within species or populations. This lack of knowledge has limited our understanding of the functional genetic variations in *OsTPP7* and their impact on AG tolerance. In order to address this gap, we analyzed 475 Korean rice accessions and conducted genetic and haplotypic analyses of *OsTPP7* (*Os09g0369400*) and its genetic backgrounds. By understanding the population genetic structure and evolutionary relationships for *OsTPP7* within and among populations, this study will be helpful in future breeding programs aimed at developing economically desirable rice varieties.

## 2 Materials and methods

### 2.1 Plant materials and experimental site

We used a core set of 475 accessions from the Korean World Rice Collection (KRICE), consisting of 421 cultivated and 54 wild accessions (**Supplementary Table 1**). The cultivated accessions, collected worldwide by the National Gene Bank of the Rural Development Administration, Republic of Korea, were classified into three varietal types: landrace, bred, and weedy (Maung et al., 2021a; Maung et al., 2021b; Phitaktansakul et al., 2022). These 421 cultivated rice accessions were further classified into six groups based on their ecotype: temperate *japonica* (279 accessions), tropical *japonica* (26 accessions), *indica* (102 accessions), aus (9 accessions), aromatic (2 accessions), and admixture (3 accessions) (Kim et al., 2007). We determined the ecotypes of the different rice accessions by utilizing whole-genome resequencing data (Phitaktansakul et al., 2022). The data was used to construct a neighbor-joining tree using PHYLIP software (Felsenstein, 2004). The resulting tree was then classified based on the previously reported ecotypes of known accessions. In addition, 54 wild rice accessions obtained from the International Rice Research Institute were included in the study (**Supplementary Table 1**). Weedy rice is an invasive and problematic type of rice that has evolved from de-domestication events from cultivated rice (He et al., 2017; Li et al., 2017). On the other hand, wild rice is a distinct species native to specific regions and is known for its unique grain characteristics (Roma-Burgos et al., 2021). All accessions were cultivated in an experimental field at the Plant Resources Department, Kongju National University (Yesan Campus), following standard crop management and cultural operations.

### 2.2 Screening for AG phenotypes

A total of 421 cultivated rice accessions were subjected to phenotypic screening for AG tolerance. To break dormancy, seeds from each accession were incubated at 30°C for one day, 48°C for three days, and then at 30°C for one day. After surface sterilization with a 1% sodium hypochlorite solution for 10 min, the seeds were washed with deionized distilled water thrice. Subsequently, the seeds were subjected to two treatments: normal germination (control) and flooded germination. Twenty-one days is a commonly used duration for AG evaluation under flooded soil conditions (Ismail et al., 2009; Ghosal et al., 2019; Kuya et al., 2019). However, *in vitro* studies have shown that this period can vary from 4-15 days depending on the purpose of investigation (Nghi et al., 2019; Islam et al., 2022; Thapa et al., 2022; Shanmugam et al., 2023). We selected a 14-day period to capture the period up to the emergence stage, which is crucial for evaluating seedling vigor and emergence, ultimately reflecting coleoptile elongation. Ten seeds of each accession were wrapped in wet absorbent filter paper for normal germination and placed in a plastic box for 14 days. For the flooded germination treatment, ten seeds from each accession were placed in a 15 ml conical tube filled with sterile water ensuring they were completely submerged, and incubated in the dark at 30°C for 14 days. The water levels of the absorbent filter paper and tubes were maintained throughout the experiment. The experiments were performed in triplicate (total 30 seeds) for both treatments, and the length of the coleoptiles was measured after 14 days of germination using an ordinary ruler. The flooding tolerance index (FTI) was calculated as the ratio of flooded coleoptile length (FCL) to normal coleoptile length (NCL). Analysis of variance (ANOVA) was performed using SPSS software to evaluate the effects of genotype and genotype × environment interactions on the trait of interest. Sources of variation were determined to assess the coefficient of variation (CV) and estimate the relationship between NCL and FCL.

### 2.3 Sequence alignment and variant calling

We utilized resequencing data from the Korean rice collection, which was generated using an Illumina HiSeq 2500 Sequencing System Platform. The cultivated Korean accessions were resequenced, with an average coverage depth of 13.5× (**Supplementary Table 1**). Among the cultivated accessions, 327 had depths greater than 10× (**Supplementary Figure 1A**). For wild accessions, the average sequencing depth was 72.2×, with a maximum depth of 143.6×, and 46 accessions had depths greater than 40× (**Supplementary Figure 1B**). The resequencing data underwent a series of processing steps, including data preparation, filtering, mapping, sorting, and variant calling. The raw data was provided in FastQ format, and missing values were removed using VCFtools (Danecek et al., 2011). BWA v0.7.15 and Samtools v1.3.1 were used to index and align the Nipponbare reference genome (IRGSP 1.0) (Li and Durbin, 2009; Li et al., 2009). Duplicate reads aligned at multiple locations were removed using PICARDv1.88 (Toolkit, 2019). Final alignment and variant calling were performed using GATK tools v4.0.1.2 (McKenna et al., 2010). The resulting variants were filtered with VCFtools to eliminate false-positive SNPs/InDels. Default settings were used for most of the software and tools employed in the analysis. The VCF file containing information of genetic variants was used to evaluate genetic variation in the *OsTPP7* gene. The sequencing data of the *OsTPP7* gene were deposited in the NCBI GenBank database with the accession numbers MZ682675-MZ683149 (**Supplementary Table 1**).

### 2.4 Principal component analysis and population structure

To assess the population structure among the different groups of rice accessions, we generated a PCA plot based on two principal components (PC1 and PC2) using TASSEL5 (Bradbury et al., 2007). The resulting variable components were visualized using the ggplot2 package in R (Wickham et al., 2016). Multidimensional scaling was employed to differentiate varietal groups (bred, weedy, landrace, and wild) based on various variables obtained from the VCF file for the *OsTPP7* gene. We used VCFtools to convert the previously called variants into plink output using the PLINK analysis toolset (Purcell et al., 2007). This conversion generated a bed file, and two additional files (.bim and.fam format) were generated using a Python script. To investigate population structure, we utilized the FastStructure package tool (Raj et al., 2014). To explore population ancestry, we estimated the number of subpopulations ranging from 2 to 7. The admixed patterns of defined populations (i.e., population structure) were analyzed using average Q-values obtained from the Pophelper analytical tool (Francis, 2017) in RStudio.

To study the evolutionary relationship of the *OsTPP7* gene among various plant species, we downloaded function-annotated protein sequences from 19 different species, available at (http://plants.ensembl.org/info/data/ftp/index.html) (**Supplementary Table 2**). These species included 12 wild rice species: *Oryza barthii*, *Oryza glaberrima*, *Oryza longistaminata*, *Oryza glumipatula*, *Oryza nivara*, *Oryza rufipogon*, *Oryza sativa japonica*, *Oryza sativa indica*, *Oryza sativa japonica, aus, Oryza meridionalis*, *Oryza punctata*, and *Oryza brachyantha*, six cereals and one dicot. We identified orthologous genes by running OrthoFinder v2.5.4 (Emms and Kelly, 2015) and generated a neighbor-joining tree using MEGA7 (Kumar et al., 2016).

### 2.5 Nucleotide diversity and evolutionary analysis

To analyze evolutionary patterns, we calculated nucleotide diversity (π), Tajima’s D, and population differentiation (*FST*) using VCFtools 0.1.13. First, we extracted variant files for the gene region of each group using VCFtools and compared them for both SNPs and InDels. Only sites with a minor allele frequency (MAF) greater than 0.05 and no missing data were included in the analysis. For nucleotide diversity and Tajima’s D tests (Tajima, 1989), we used a sliding window size of 1.0 kb and compared the results among the different groups of the 475 rice accessions. *FST* was also calculated using VCFtools v0.1.15, with a 500-bp slide window and 500-bp steps, to assess genetic differences among the groups.

### 2.6 Haplotype analysis

Furthermore, we performed a haplotype analysis on the *OsTPP7* gene region to group its functional variations (SNPs and InDels). VCFtools was used to create a FASTA file from the VCF file, and sequences were aligned using MEGA7 (Kumar et al., 2016). The aligned sequences were then transformed to the nexus format before conducting haplotype analysis with DnaSP v6.12.03 (Rozas et al., 2017). We constructed a TCS haplotype network (Crandall et al., 2000) using the Population Analysis with Articulate Tree (PopART) v1.7 software (Leigh and Bryant, 2015). Additionally, we conducted haplotype analysis in the 3K_RG panel for the *OsTPP7* gene. The sequence data for the 3K_RG panel was downloaded from the online database https://snp-seek.irri.org/_download.zul;jsessionid=79B65E769E27730C020C4C198AE67235 accessed on May 22, 2020, and haplotyping was performed without filtering the variants.

### 2.7 Statistical analysis

All statistical analyses were performed using R Statistical Software (version 4.2.3; R Foundation for Statistical Computing, Vienna, Austria). The results are presented as the mean ± standard error (SE). One-way ANOVA and Sheffe’s test were used to detect significant differences. Further statistical comparisons were conducted to determine the associations between the identified functional haplotypes and AG tolerance using the phenotypic data from the cultivated accessions. The major haplotypes were used for comparison, and the mean phenotype was calculated for each group of accessions carrying a similar haplotype. The difference between the means was compared by the Student’s t-test using the ‘t.test’ function in the R.

## 3 Results

### 3.1 Phenotypic variation for AG

The cultivated rice accessions from the Korean collection showed considerable variation in AG phenotyping, as summarized in **Table 1**. Under normal conditions, the NCL ranged from 0.42 cm to 3.85 cm, with a mean of 1.94 cm. Under flooded conditions, the FCL ranged from 0.69 cm to 5.37 cm, with a mean of 2.80 cm. *Japonica* accessions displayed higher coleoptile lengths (CLs) than *indica* accessions under both normal and flooded conditions. The average CLs for *indica*, temperate *japonica*, and tropical *japonica* ecotypes under normal germination were 1.92 cm, 2.07 cm, and 1.99 cm, respectively. These values increased in response to AG, resulting in average CLs of 2.26 cm, 3.02 cm, and 2.89 cm, respectively. Consequently, the FTI values were also higher in *japonica* compared to *indica* (**Table 1**).

**Table 1:** Statistical analysis of rice coleoptile lengths from 421 cultivated rice accessions under normal and flooded conditions.

Source of variables	NCL (cm)	FCL (cm)	FTI
*Indica*	1.92 (0.45-3.11)	2.26 (0.69-3.59)	1.30 (0.29-4.57)
Temperate *japonica*	2.07 (0.42-3.85)	3.02 (1.11-5.37)	1.56 (0.41-6.61)
Tropical *japonica*	1.99 (0.50-2.74)	2.89 (1.24-4.63)	1.59 (0.71-3.95)
Admixture	2.13 (1.82-2.49)	2.84 (2.42-3.08)	1.37 (0.97-1.70)
Aromatic	1.93 (1.54-2.32)	2.26 (1.92-2.60)	1.26 (0.83-1.68)
Aus	2.17 (1.75-2.84)	1.26 (1.00-2.29)	0.59 (0.45-1.31)
Mean ± SE	2.03 ± 0.029	2.80 ± 0.044	1.48 ± 0.033
Range	0.42–3.85	0.69–5.37	0.29–6.61
Std. Deviation	0.54	0.84	0.62
CV %	30.02	32.42	46.23
G × E	***	***	***
Genotype	***	***	***
Environment	^ns^	^ns^	^ns^

NCL, normal coleoptile length; FCL, flooded coleoptile length; FTI, flooding tolerance index; Values in parenthesis indicate a range; ± SE, standard error; CV, coefficient of variation; G × E, genotype by environment and *** = p < 0.0001 and ns, non-significant difference.

The ANOVA revealed that the interaction effect between genotype and environment strongly influenced coleoptile lengths (**Table 1**). Additionally, the genotypic effect was significant for all traits, while the environmental effect was not significant. The coefficient of variation (CV) for FTI was 46.23%, higher than that for NCL (30.02%) and FCL (32.42%). The standard deviation was also higher for FCL (0.90) compared to NCL and FTI (**Table 1**).

**Figure 1:**
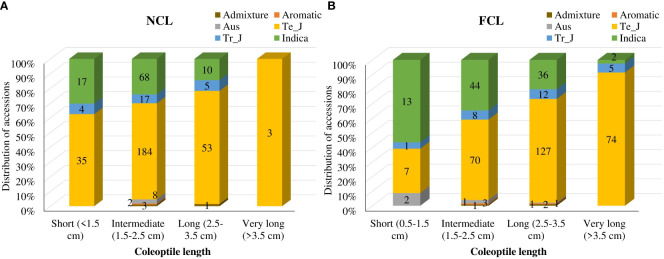
Classification of coleoptile lengths under normal **(A)** and flooded conditions **(B)**. Numbers on the bars indicate total accessions. Te_J represents temperate *japonica*, and Tr_J represents tropical *japonica*.

Furthermore, we categorized the distinct variations in coleoptile traits into four groups based on length: short (<1.5 cm), intermediate (1.5-2.5 cm), long (2.5-3.5 cm), and very long (> 3.5 cm) (**Figure 1**; **Supplementary Figure 2**). Interestingly, the number of accessions in the short and intermediate categories decreased, while the number in the long and very long categories increased under AG. Under normal conditions, 68 indica accessions were grouped in the intermediate category, but only 44 remained in that category under AG. Whereas the number of accessions in the long category increased from 10 to 36 under normal and AG conditions, respectively. Similar trends were observed for temperate *japonica* and tropical *japonica* accessions. While only three accessions had NCL >3.5 cm, 81 accessions showed very long FCL under AG (**Figure 1**).

**Figure 2:**
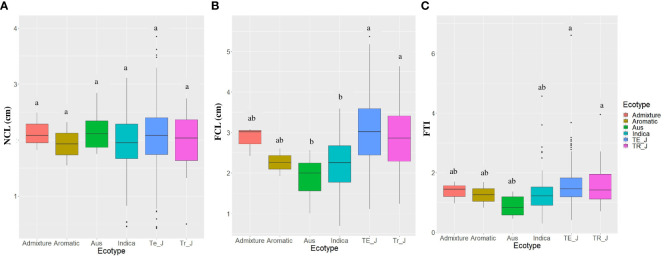
Variations in anaerobic germination (AG) tolerance traits among the ecotypes. **(A)** NCL, normal coleoptile length; **(B)** FCL, flooded coleoptile length; **(C)** FTI, flooding tolerance index; SEM error bars are displayed for each type; Te_J represents temperate *japonica*, and Tr_J represents tropical *japonica*. Different letters above each boxplot indicate significant differences among ecotypes according to Sheffe’s test (*p* < 0.05).

Regarding the different ecotypes, no significant differences were found in NCL. However, significant variations were observed in FCL and FTI between the ecotypes. Notably, both temperate *japonica* and tropical *japonica* ecotypes exhibited significantly higher FCL and FTI values compared to the *indica* and aus ecotypes (**Figure 2**). These findings underscore the differential response of temperate *japonica* and tropical *japonica* ecotypes towards AG, as evidenced by their significantly higher FCL compared to the *indica* ecotypes.

### 3.2 Genetic variation in *OsTPP7* of the Korean rice collection

The *OsTPP7* gene (*Os09g0369400*) is located on chromosome 9 at 12,251,875.12,254,061 (+ strand) and consists of seven exons with a length of 2186 bp. **Table 2** presents the genetic variants, including SNPs, insertions (Ins), and deletions (Del), in the *OsTPP7* gene region of the 475 rice accessions. Among the cultivated group, temperate *japonica* had 8 SNPs, followed by six SNPs in *indica*. In contrast, no genetic variation was observed in the aromatic group, which matches the Nipponbare reference sequence. A total of 163 genetic variations (53 Ins, 33 SNPs, and 23 Del) were identified in wild rice.

**Table 2:** Summary of genetic variations in the *OsTPP7* gene region of 475 accessions from the Korean rice collection.

Group	Ecotypes	Total No. of variations			No. of Accessions
		SNPs	Ins	Del	
Cultivated rice	Temperate *japonica*	8	2	3	279
	Tropical *japonica*	1	0	0	26
	*Indica*	6	3	3	102
	Aus	5	3	2	9
	Aromatic	0	0	0	2
	Admixture	5	2	3	3
Wild rice	*Oryza rufipogon*	1	1	0	3
	*Oryza nivara*	5	3	2	3
	Other wild rice	27	49	21	48

SNPs, Single Nucleotide Polymorphisms, Ins, insertion, Del, deletion, and Other wild rice, a group of wild rice accessions excluding *Oryza rufipogon* and *Oryza nivara*.

### 3.3 Population structure analysis

**Figure 3:**
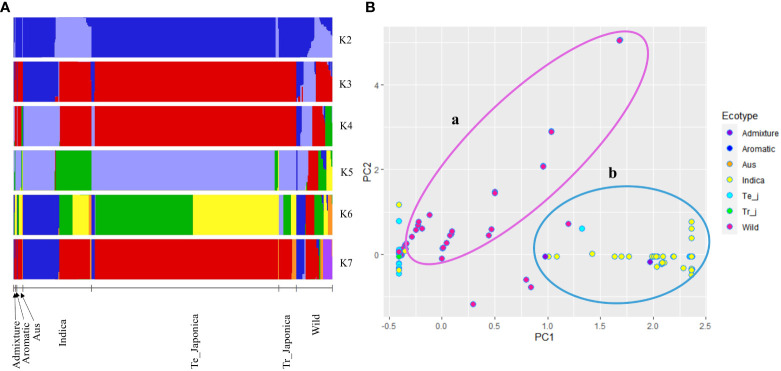
Population structure of 475 rice accessions based on the *OsTPP7* gene. **(A)** Population structure clustering using K values ranging from 2 to 7; each color represents a cluster. **(B)** Principal component analysis (PCA) of cultivated and wild rice accessions. Circle a: distribution of wild accessions, and circle b: distribution of cultivated accessions.

The genetic composition of the Korean rice collection was further examined for *OsTPP7* gene region using population structure and PCA (**Figure 3**). It was observed that the *indica* and *japonica* ecotypes exhibited a somewhat similar structure at K = 3, K = 4, and K = 7. Interestingly, the *indica* ecotype was divided into two clusters, representing temperate and tropical *japonica*, indicating a certain degree of genetic similarity between *indica* and one or both of these ecotypes in relation to the *OsTPP7* gene. In contrast, wild rice exhibited a mixed structure that became more apparent with increasing K values (**Figure 3A**). Similarly, in the PCA, the wild rice accessions exhibited a scattered distribution (part a in **Figure 3B**), while some overlap was observed among the cultivated groups (part b in **Figure 3B**). Furthermore, PCA analysis based on varietal type also revealed a similar dispersion pattern for bred, landrace, weedy, and wild accessions (**Supplementary Figure 3A**).

Our analysis of orthologous genes revealed distinct sub-clades formed by all *TPP* gene members, with *TPP7* genes clustered together with *TPP6* (**Supplementary Figure 4**). Furthermore, in the PCA analysis, we observed clear differentiation of wild rice accessions from other ecotypes based on the first and second principal components (PC1 and PC2) (**Figure 3B**), which aligns with previous studies (Veasey et al., 2011; Huang et al., 2012). These findings suggest that wild rice may possess a larger genetic distance and a different genetic background, indicating a complex domestication history during the evolution of wild rice (Konishi et al., 2006; Choi et al., 2019).

### 3.4 Genetic differentiation for *OsTPP7* in Korean rice collection

To assess the level of differentiation among the populations, we calculated fixation index (*FST*) values based on the *OsTPP7* gene using weighted methods (Weir and Cockerham, 1984). The resulting statistics were then analyzed for pairwise comparisons (**Table 3**). The highest weighted average *FST* (0.533) was observed between the temperate *japonica* and wild groups, while the lowest was observed between the temperate *japonica* and aromatic groups (**Table 3**). Interestingly, the wild group showed a relatively closer genetic relationship to the aromatic and admixture groups.

**Table 3:** Pairwise estimates of genetic differentiation (*F_ST_*) values among the ecotypes for the *OsTPP7* gene region.

Ecotypes	*Indica*	Te_J	Tr_J	Aromatic	Aus	Admixture
Te_J	0.261					
Tr_J	0.257	0.122				
Aromatic	0.00	0.00	0.003			
Aus	0.103	0.104	0.106	0.00		
Admixture	0.00	0.274	0.318	0.007	0.062	
Wild	0.271	0.533	0.211	0.000	0.097	0.00

For the *indica* group, the highest pairwise *FST* value was observed with wild type (0.271), followed by temperate *japonica* (0.261), while lower genetic differentiation was observed for the aromatic and admixture groups. The *FST* values between the tropical *japonica* and admixture groups (0.318) and between the temperate *japonica* and admixture groups (0.274) indicated a higher degree of genetic differentiation among the cultivated population (**Table 3**).

Regarding varietal types, *FST* values ranged from 0.003 (between bred and weedy) to 0.459 (between bred and wild). These results revealed that pairwise *FST* values between cultivated groups were lower than those involving the wild group, indicating a closer genetic relationship for *OsTPP7* within the cultivated varietal types (**Supplementary Figure 3B**).

### 3.5 Genetic diversity, selection, and demographic history of *OsTPP7* in Korean Rice collection

**Figure 4:**
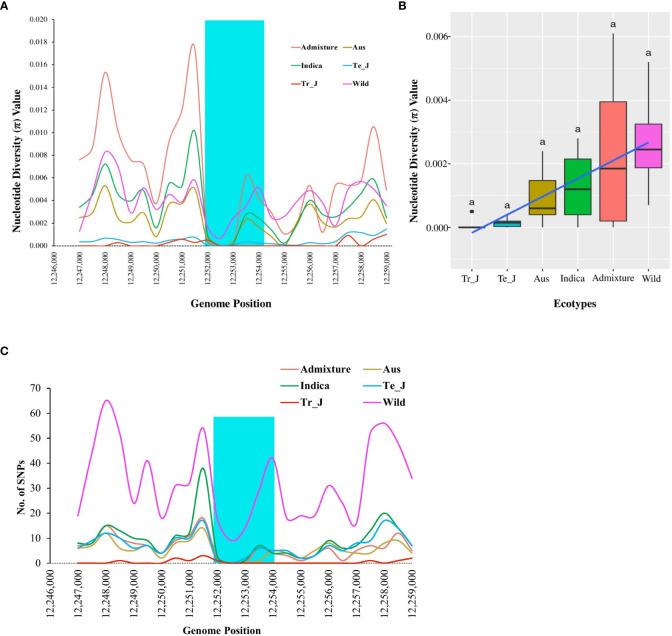
Nucleotide diversity based on *OsTPP7* across ecotypes of Korean rice collection. **(A)** Nucleotide diversity values with 1.0 kb sliding window. The highlighted cyan color shows the *OsTPP7* gene region. **(B)** Box plots represent a comparison of mean nucleotide diversity among the ecotypes. Different letters above each boxplot indicate significant differences among ecotypes according to Sheffe’s test (*p* < 0.05). **(C)** Variation in SNP density among the ecotypes of Korean rice collection for *OsTPP7* gene region.

The nucleotide diversity (π) for the *OsTPP7* gene was analyzed to evaluate the degree of polymorphism among different groups within the Korean rice collection. The results revealed that the wild group had the highest diversity, followed by admixture, *indica*, aus, temperate-*japonica*, and tropical-*japonica* groups (**Figure 4A**). On average, the nucleotide diversity was 0.00008 in tropical *japonica* and 0.00265 in wild rice (**Figure 4B**). Notably, the highest value of nucleotide diversity (0.00515) was observed at position 12,254,000 in the wild group, while the weedy group had the lowest value (0.00042) at the same position (**Supplementary Figure 5A**). When considering the mean nucleotide diversity values, the wild group had the highest value (0.00310), followed by landrace (0.00219), bred (0.00131), and weedy (0.00086) (**Supplementary Figure 5B**). The diversity of *japonica* in this gene region was lower than that of *indica* and other groups, supporting the hypothesis of selection for *OsTPP7* during the domestication of *japonica* rice.

**Figure 5:**
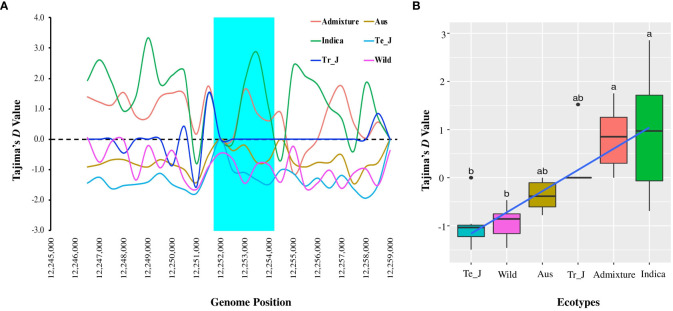
Tajima’s *D* analysis based on *OsTPP7* in ecotypes of Korean rice collection. **(A)** Tajima’s *D* values among the ecotypes with a 1.0 kb sliding window. The highlighted cyan color indicates the *OsTPP7* gene region. **(B)** Box plots comparing the mean Tajima’s D values among the ecotypes. Different letters above each boxplot indicate significant differences among ecotypes according to Sheffe’s test (*p* < 0.05).

To gain further insights into the presence of selection and/or demographic changes within the population, we analyzed Tajima’s D values for the *OsTPP7* gene and assessed the differences between the expected and observed segregation numbers due to selection. Here, *indica* type showed a positive value (0.97190), followed by the admixture (0.6003) and tropical *japonica* (0.21739) ecotypes, while negative values were observed in temperate *japonica*, aus, and wild types (**Figure 5**). For varietal types, the average Tajima’s D values ranged from −0.94356 (wild) to 0.00020 (landrace), with only the landrace showing a positive value (0.00020) (**Supplementary Figure 6**). The positive Tajima’s D values observed in the *indica*, admixture, and tropical *japonica* ecotypes suggest the presence of balancing selection or population contraction. In contrast, the negative Tajima’s D values observed in the temperate *japonica*, aus, and wild types suggest a deficiency of intermediate-frequency variants, which could be due to purifying selection or population expansion.

### 3.6 Haplotype diversity analysis of *OsTPP7* gene

**Figure 6:**
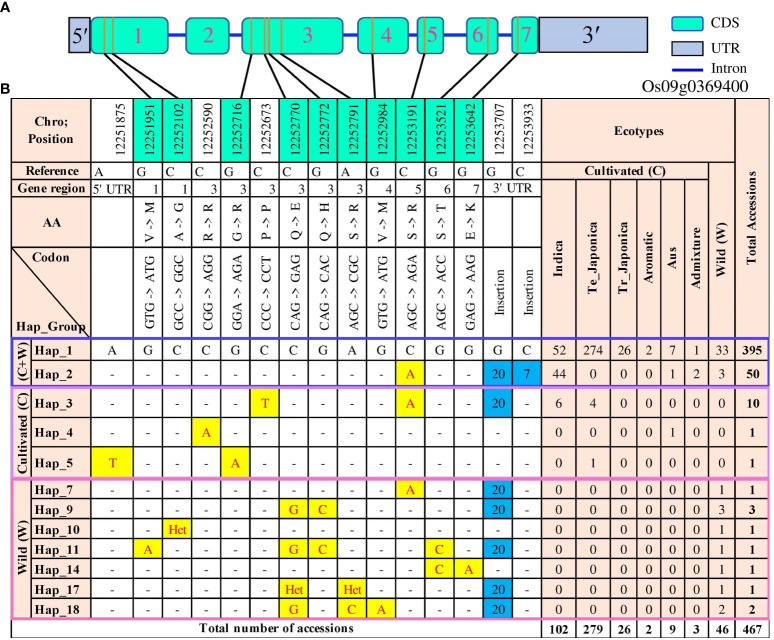
Gene structure and functional haplotypes of *OsTPP7* (*Os09g0369400*) in 475 accessions. **(A)** The gene structure of the *OsTPP7* gene was generated using Gene Structure Display Server (GSDS v2.0) program (http://gsds.gao-lab.org/). **(B)** Distribution of functional SNPs and haplotypes. Different SNPs are highlighted in yellow, insertions are highlighted in blue, and the dash (-) represents the same SNP. “Het” refers to “heterozygote,” and “C + W” refers to both cultivated and wild groups. The cyan color in chromosome position shows the functional SNPs.

In order to investigate the genetic diversity and relationships among different haplotypes of the *OsTPP7* gene, a haplotype network was constructed (**Figure 6A**). A total of 92 polymorphic sites, including 34 SNPs and 58 InDels, were detected within the *OsTPP7* genic region. These variations were distributed across different regions, with 23 sites in the exon region, 43 sites in introns, four sites in the 5′UTR, and 22 sites in the 3′UTR (**Supplementary Table 3**). The haplotype analysis revealed 18 distinct haplotypes, with three specific to cultivated accessions, 13 specific to wild accessions, and two present in both cultivated and wild accessions. The most common haplotype (Hap_1) was found in 395 rice accessions, including 362 cultivated and 33 wild accessions (**Supplementary Table 4**). Notably, among 305 *japonica* accessions, 300 were *japonica* accession into Hap_1, while Hap_2 and Hap_3 were predominated associated with indica accessions (**Figure 6**). A closely connected network was observed among the haplotypes of cultivated rice accessions, in which Hap_2, Hap_4, and Hap_5 were derived from the major haplotype, Hap_1, suggesting their close relationship (**Supplementary Figure 7**). Conversely, the wild rice haplotypes formed a network with varying degrees of mutational steps, clearly demonstrating a considerable genetic distance between cultivated and wild rice (**Supplementary Figure 7**).

Within the coding region of *OsTPP7*, we identified ten functional (non-synonymous) SNPs (referred to as fSNPs hereafter). One of these fSNPs was a C/A substitution at position 12,253,191 in exon 5, resulting in serine to arginine change found in Hap_2. This haplotype was present in 50 accessions, including 44 *indica*, one aus, two admixture, and three wild accessions. Another haplotype, Hap_3, was detected in six *indica* and four temperate *japonica* accessions. Hap_3 was characterized by a non-functional C/T SNP at position 12,252,673 in exon 3, a functional C/A SNP at position 12,253,191 in exon 5, and a 20-bp insertion in the 3’ UTR region. Additionally, Hap_5, found in one temperate *japonica* accession, had an fSNP G/A at position 12,252,716, causing a substitution from glycine to arginine in exon 3. The most prevalent haplotype, Hap_1, was present in temperate *japonica* (274 accessions), *indica* (52 accessions), aus (seven accessions), wild (33 accessions), admixture (one accession), tropical *japonica* (26 accessions), and aroma rice (two accessions) (**Figure 6**).

Among the wild group, haplotype analysis identified 15 haplotypes, nine of which contained fSNPs. Hap_10 had one heterozygote functional allele, while the remaining eight fSNPs (including one C/A, three G/A, two C/G, and two G/C) were unique to the wild type. Hap_9, present in three wild accessions, had two fSNPs (C/G and G/C), while Hap_18, found in two accessions, had a G/A fSNP causing a valine to methionine substitution in exon 4 (**Figure 6**).

Furthermore, we analyzed 3,000 rice genome (3K-RG) data to evaluate the *OsTPP7* gene polymorphisms in a large number of accessions. A total of 112 haplotypes were identified, consisting of 105 polymorphisms, including 100 SNPs and 5 InDels. Among these, 72 SNPs were fSNPs located in the coding region, while the 5 InDels (two insertions and three deletions) were present in the intron and 3’ UTR (**Supplementary Tables 5**, **6**). The primary haplotype Hap_1 was detected in 1,485 accessions, followed by Hap_2 in 1,107 accessions. Hap_2 was characterized by a C/A fSNP at position 12,253,191, causing a serine to arginine substitution in exon 5. Another major haplotype, Hap_5, was found in 223 accessions and featured the fSNP G/T at position 12,252,094 in exon 1. Hap_7 exhibited a 20 bp insertion in the 3’ UTR region and was characterized by the fSNP C/A at position 12,253,191 and the non-functional SNP C/T at position 12,252,673 (**Supplementary Figure 8**).

### 3.7 Association between *OsTPP7* haplotypes and coleoptile length

**Figure 7:**
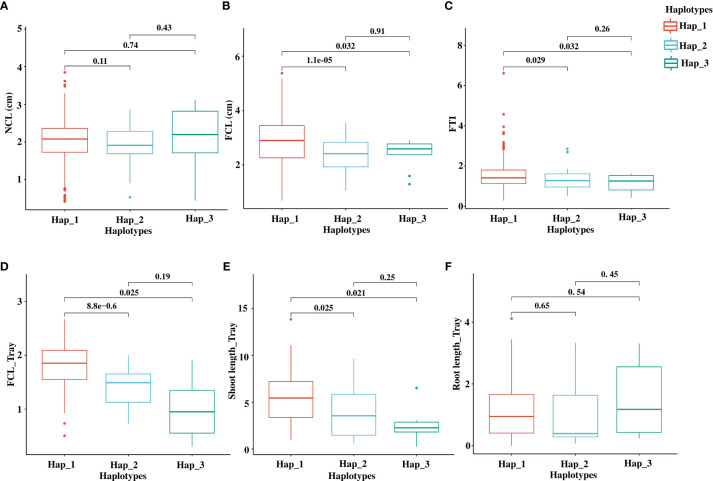
The phenotypic effect of *OsTPP7* haplotypes on AG traits. Boxplot shows the impact of haplotypes on NCL **(A)**, FCL **(B)**, and FTI **(C)** from a conical tube-based experiment. The boxplot also shows the impact of haplotypes on FCL **(D)**, shoot length **(E)**, and root length **(F)** from a tray-based experiment. All lengths are in cm. The significant difference between haplotypes was tested with *p* < 0.05 based on t-test statistics.

Next, we evaluated the phenotypic performance of the major haplotypes, Hap_1, Hap_2, and Hap_3, across cultivated accessions (362 accessions in Hap_1, 47 accessions in Hap_2, and ten accessions in Hap_3) (**Figures 7A–C**). By conducting pairwise t-tests at a significance level of 0.05, we observed significant differences in FCL between Hap_1 and the other haplotypes, Hap_2, and Hap_3 (**Figure 7B**). For FTI, significant differences were observed between Hap_1 and Hap_2/Hap_3, although the significance levels were relatively lower compared to FCL (**Figure 7C**). Hap_2, an *indica*-specific haplotype with 44 *indica* accessions, displayed lower FCL and FTI values than Hap_1. However, there was no significant variation in NCL among the haplotypes (**Figure 7A**).

To further validate the findings from the conical tube experiment and provide additional evidence for the impact of *OsTPP7* haplotypes on AG tolerance, we conducted a tray-based experiment for AG phenotyping. We randomly selected a total of 137 accessions from three major haplotypes, including Hap_1 (107 accessions), Hap_2 (23 accessions), and Hap_3 (six accessions). AG phenotyping was carried out based on the protocol of Septiningsih et al. (Septiningsih et al., 2013) (**Supplementary Methods**). The results revealed significant differences between Hap_1 and Hap_2/Hap_3 for FCL and shoot length (**Supplementary Table 7**; **Figures 7D, E**). These findings provide further support for the notion that allelic variations within *OsTPP7* contribute to the phenotypic variation in coleoptile response to anaerobic germination rather than normal germination.

## 4 Discussion

The functional significance of the *OsTPP7* gene, which is expressed during the early stages of coleoptile elongation and plays a role in AG tolerance, has been relatively understudied in rice. Early-season flooding poses a significant challenge in direct-seeded rice (DSR) as different rice genotypes exhibit varying coleoptile lengths during anoxia. Although rice is semi-aquatic and adapted to a wide range of hydrological conditions, it has a certain degree of tolerance to insufficient oxygen for AG and anaerobic seedling development. Under limited oxygen conditions, rice can use the starchy reserves available in the endosperm, and rice varieties expressing the *TPP7* gene are more efficient at transporting sugars from the source (endosperm) to the sink (embryo and coleoptile) compared to other crops (Pucciariello, 2020). In order to sustain growth, rice seeds express the molecular signaling cascade involving the CIPK15-SnRK1A-MYBS1-αAmy pathway, which is responsible for mobilizing reserves and promoting growth (Kretzschmar et al., 2015). This pathway is initiated by the activation of CIPK15 (calcineurin b-like interacting protein kinase 15), which subsequently activates SnRK1 (sensor sucrose nonfermenting 1-related protein kinase 1), a regulatory kinase that governs the transcription factor MYBS1 (myeloblastosis sugar response complex 1). MYBS1 then translocates to the nucleus and binds to sugar-responsive elements on the promoters of α-amylases, particularly the predominant isoform α-amylase 3D (RAMY3D) (Lasanthi-Kudahettige et al., 2007; Lee et al., 2009). In this process, *OsTPP7* plays a crucial role by disrupting the T6P/sucrose homeostasis and preventing the repression of the CIPK15-SnRK1A-MYBS1-αAmy pathway. By facilitating the activation of α-amylase expression in the embryo-coleoptiles, *OsTPP7* contributes to effective coleoptile elongation and supports the growth and development of rice seedlings (Magneschi and Perata, 2009; Kretzschmar et al., 2015; Nghi et al., 2019; Yu et al., 2021). Using flooding-tolerant cultivars is the most suitable method to protect rice yields during prolonged flooding (Angaji et al., 2010). Tolerant rice cultivars exhibit rapid elongation of their coleoptiles, enabling seedlings to escape the anoxic environment and increasing their chances of survival (Yang et al., 2019). *Japonica* rice varieties have been observed to elongate their coleoptiles faster than *indica* varieties under flood conditions (Hsu and Tung, 2015). However, the full potential of local rice germplasms for germination and seedling development under flooded conditions remains largely untapped (Illangakoon et al., 2019).

Recognizing the significance of AG, we evaluated the coleoptile length of a diverse rice panel under normal and flooded conditions to assess their AG tolerance. Our findings indicated that most accessions showed enhanced coleoptile lengths under flooded conditions compared to normal conditions, with temperate *japonica* exhibiting the highest mean values for both NCL and FCL (**Table 1**). This indicates that temperate *japonica* has a flooding escape type of tolerance, which is consistent with previous studies highlighting its superior coleoptile elongation compared to other rice ecotypes (Kuya et al., 2019). Coleoptile elongation is considered as a major response to anaerobic stress, enabling direct sowing instead of transplanting and thereby improving the economic sustainability of rice cultivation. In this context, the qAG-9-2 QTL, which contains a functional *TPP7* gene associated with coleoptile elongation, represents a promising avenue for improving flooding tolerance during germination (Kretzschmar et al., 2015). Despite its potential, limited research has explored the relationship between flooding tolerance and haplotypes of candidate genes in rice, particularly the *OsTPP7* gene. Screening over 8,000 accessions from the International Rice Research Institute has revealed only a few genotypes with strong germination ability under flooding conditions, emphasizing the importance of exploring and harnessing the potential of local rice germplasms for germination and seedling development in flooded environments (Angaji et al., 2010).

The genetic diversity indices of the *OsTPP7* gene reveal variation in nucleotide diversity and Tajima’s D values among rice ecotypes and varietal types (**Figures 4**, **5**). The nucleotide diversity (π) analysis indicates that the wild group exhibited the highest diversity, followed by the admixture, *indica*, aus, temperate-*japonica*, and tropical-*japonica* ecotypes. This suggests distinct selection and purification for *OsTPP7* among these ecotypes, with *japonica* exhibiting lower heterozygosity. This finding aligns with previous reports by Rashid and Zhao et al., who observed lower nucleotide diversity (π) in *japonica* than *indica*, but higher diversity in wild rice (Rashid et al., 2016). Among the varietal types, the highest nucleotide diversity (π) was observed in wild rice (**Supplementary Figure 5**), indicating the retention of rich ancestral genetic variation within the existing population (Yu et al., 2011; Deng et al., 2020). Lower nucleotide diversity (π) among the tested populations or samples may be attributed to inbreeding depression, increased genetic drift, and ineffective selection processes (Teixeira and Huber, 2021). The *F_ST_* analysis of the *OsTPP7* gene showed that two major varietal types, wild and bred, were isolated by the *F_ST_* value 0.459, and there was a close genetic distance between the bred and weedy groups (0.003) (**Supplementary Figure 3B**). Significant genetic differentiation was identified among ecotypes based on the range of *F_ST_* values (**Table 3**), with wild rice exhibiting higher genetic differentiation compared to other ecotypes (Melaku et al., 2013). The Tajima’s D analysis showed that *indica*, admixture, and tropical-*japonica* groups had positive values, while the temperate-*japonica*, aus, and wild types had negative values (**Figure 5**). The landrace group had a positive Tajima’s D value, suggesting it may have experienced balancing selection or population subdivision for the *OsTPP7* gene. Ecotypes with negative Tajima’s D values are expected to have undergone purifying selection or selective sweeps on the *OsTPP7* gene. Positive Tajima’s D values arise from an excess of intermediate frequency alleles and can result from population contraction or balancing selection (Tajima, 1989). Overall, these results suggest that different selection pressures and demographic events across different rice ecotypes and varietal types have influenced the genetic diversity of the *OsTPP7* gene.

Haplotypes play a crucial role in both the imputation process and selection signature analysis, and their size is influenced by recombination events within a population (Mészáros et al., 2021). Identification and deployment of functional haplotypes in breeding programs have gained attention as a promising approach known as haplotype-based breeding (Bhat et al., 2021; Rana et al., 2022). In this study, we conducted a comprehensive haplotype analysis of the *OsTPP7* gene and identified 18 haplotypes, including ten functional and eight non-functional haplotypes, based on 92 polymorphic sites within the genic region (**Supplementary Table 3**). Notably, we discovered a novel haplotype, Hap_2 (with fSNP C/A), which was detected in both the Korean rice collection (50 accessions) and the 3k_RG (1,107 accessions), highlighting its potential relevance for the future breeding of AG-tolerant rice. The lower FCL, FTI, and shoot length associated with Hap_2 in Korean rice accessions highlights its reduced AG tolerance (**Figure 7**). Although this haplotype may not be desirable for developing AG-tolerant rice varieties, it provides an opportunity to investigate the underlying mechanisms and genetic factors associated with anaerobic germination. In addition, we identified InDel haplotypes with a 20 bp insertion at 12,253,707 and a seven bp insertion at 12,253,933 in both the 475 Korean rice core and the 3K_RG. **Supplementary Table 6** provides detailed information on the haplotypes and the corresponding accessions from the 3K_RG dataset.

Furthermore, we performed an association analysis between functional haplotypes and phenotypic variations to test their responses to coleoptile elongation. The major haplotypes exhibited significant differences in FCL, with Hap_1, which is identical to the AG-tolerant cultivar Nipponbare (Hsu and Tung, 2015), serving as the reference haplotype. Among the 305 *japonica* accessions, 300 harbored Hap_1, while 52 out of the 102 *indica* accessions shared the same haplotype (**Figure 6B**). Seven aus and two aromatic accessions also possessed Hap_1, while four temperate *japonica* accessions carried the *indica*-specific haplotype, Hap_2. Accessions with Hap_1 exhibited significantly higher FCL compared to Hap_2 and Hap_3 (**Figures 7B, D**). On the contrary, no significant associations were observed between haplotypes for NCL, indicating the independent genetic control of CL under normal and flood conditions (**Figure 7A**). The lower FCL values in the *indica* type suggest that it may have a quiescence flooding resistance type. *Indica* accessions carrying Hap_1 could be a valuable resource for improving anaerobic germination. Significant differences were also observed in shoot length between Hap_1 and Hap_2/3 (**Figure 7E**). The higher shoots length in Hap_1 may enhance trehalose accumulation in germinating coleoptiles, leading to the activation of α-AMYLASE (AMY) genes associated with endospermic starch catabolism and early elongation growth (Kretzschmar et al., 2015). At the same time, non-significant variations in root length (**Figure 7F**) suggest that rice can germinate anaerobically in flooded soils by prioritizing coleoptile and shoot elongation over the development of roots. However, the complex nature of the AG trait, which involves critical processes such as starch breakdown, glycolysis, fermentation, and various biochemical and metabolic processes, could account for subtle variations in FCL within haplotypes under flooded conditions (Singh et al., 2017; Ma et al., 2020). Further studies are warranted to gain a better understanding of AG tolerance and post-flood crop establishment. Efforts should be directed toward bridging the gap between AG-associated haplotypes and the breeding of direct-seeded rice (DSR) varieties

## 5 Conclusion

This study aimed to investigate the *OsTPP7* (*Os09g0369400*) gene, which plays a critical role in rice anaerobic germination (AG) tolerance. To gain a better understanding of its gene function, we analyzed various diversity indices across different subpopulations of rice species, including haplotype diversity, nucleotide diversity, Tajima’s D, fixation index, population structure, and PCA. Through the genetic diversity and evolutionary analysis of OsTPP7, we gained insights into its domestication signature during gene evolution. The results revealed evidence of different directional selections, as indicated by positive and negative Tajima’s D values in cultivated rice, suggesting selective sweeps in this gene. A total of 18 haplotypes were identified in the Korean rice collection, of which three major haplotypes. Further phenotypic performance of major haplotypes showed significant differences in flooded coleoptile length, flooding tolerance index, and shoot length between Hap_1 and Hap_2/3. Specifically, the majority of *japonica* accessions belonging to Hap_1 exhibited higher AG tolerance compared to Hap_2/3, which were predominantly associated with *indica* accessions. The functional haplotypes identified in the Korean rice collection and 3K_RG data could be a valuable resource for haplotype-based breeding to improve anaerobic germination tolerance in rice. These findings provide valuable information for future selective rice breeding programs and develop more efficient and effective breeding strategies.

## Data availability statement

The datasets presented in this study can be found in online repositories. The names of the repository/repositories and accession number(s) can be found in the article/**Supplementary Material**.

## Author contributions

Y-JP and S-HC acquired the funds and resources, designed the experiment, and administered and supervised the project; KA and Y-JP conceptualized the project; KA, WO, M-HM, and AS designed the methodology and performed the experiments; KA, TM, and JN curated the data, and performed the formal analysis; KA and BN wrote the draft manuscript; S-HC and K-WK Resources; K-WK, BN, S-HC, and C-YL writing -review and editing. All authors contributed to the article and approved the submitted version.
