# Supplementary material for: Genomic landscape of the OsTPP7 gene in its haplotype diversity and association with anaerobic germination tolerance in rice
Source: Front Plant Sci. 2023 Jul 25;14:1225445. doi: 10.3389/fpls.2023.1225445 (PMC10407808; doi:10.3389/fpls.2023.1225445)
Supplement: Supplementary file 1 [file DataSheet_1.zip › Figures S1 - S8.docx]

Supplementary Material

**Genomic landscape of the *OsTPP7* gene in its haplotype diversity and association with anaerobic germination tolerance in rice**

Kyaw Myo Aung ^1^, Win Htet Oo ^1^, Thant Zin Maung ^1^, Myeong-Hyeon Min ^1^, Aueangporn Somsri ^1^, Jungrye Nam^2^, Kyu-Won Kim ^2^, Bhagwat Nawade ^2^, Chang-Yong Lee^3^, Sang-Ho Chu ^2,*^_,_ and Yong-Jin Park ^1,2,*^

*** Correspondence:**

Sang-Ho Chu (sanghochu76@gmail.com)

Yong-Jin Park (yjpark@kongju.ac.kr)

**Supplementary method:**

A tray-based experiment was conducted to validate the results obtained from the conical tube experiment and to provide additional evidence for the impact of *OsTPP7* haplotypes on AG tolerance. A total of 137 accessions were randomly selected from three major haplotypes, including Hap_1 (107 accessions), Hap_2 (23 accessions), Hap_3 (six accessions), and the phenotypic screening for AG was carried out following the protocol of (Septiningsih et al., 2013). Ten dry seeds from each accession were sown in seedling trays in a greenhouse, where the average high temperature was maintained at 30°C and the low temperature at 26°C. The trays were submerged in 10 cm of tap water, and three replications were used with 30 seeds in each accession. After 14 days of sowing, the coleoptile length, number of shoots, long shoot length, and number of roots were measured to evaluate the effects of *OsTPP7* haplotypes on AG tolerance.

**Supplementary Figures**


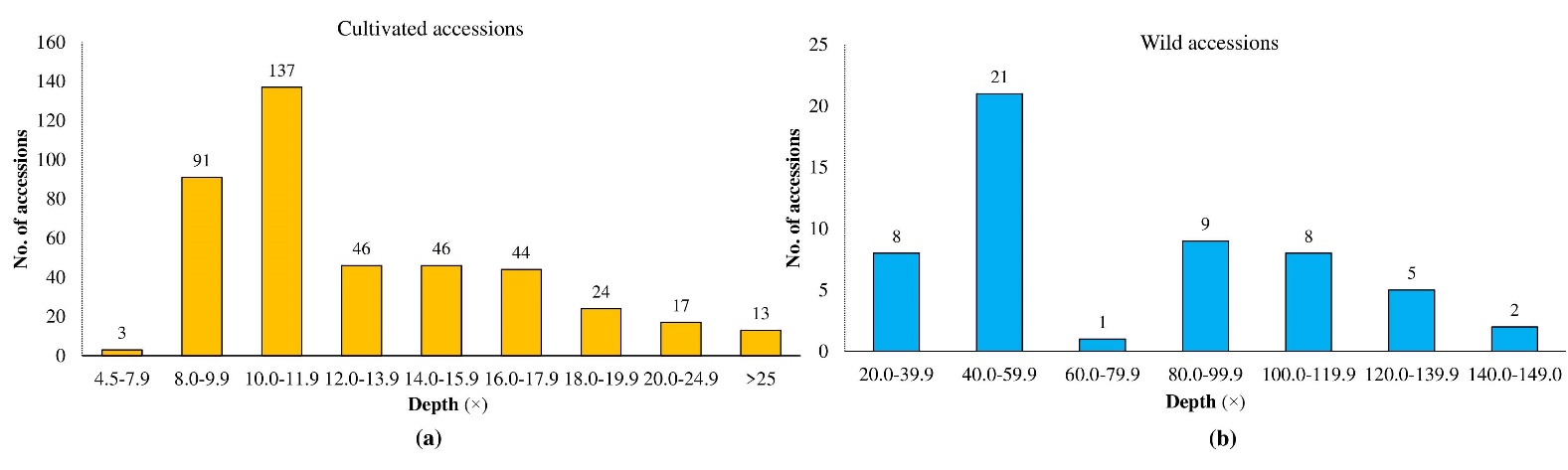


**Supplementary Figure 1.** Details on resequencing depths (×) of Korean rice collection. (a) Depths (×) of 421 cultivated accessions (b) Depths (×) of 54 wild accessions. The total number of accessions in each category is shown on the bar.


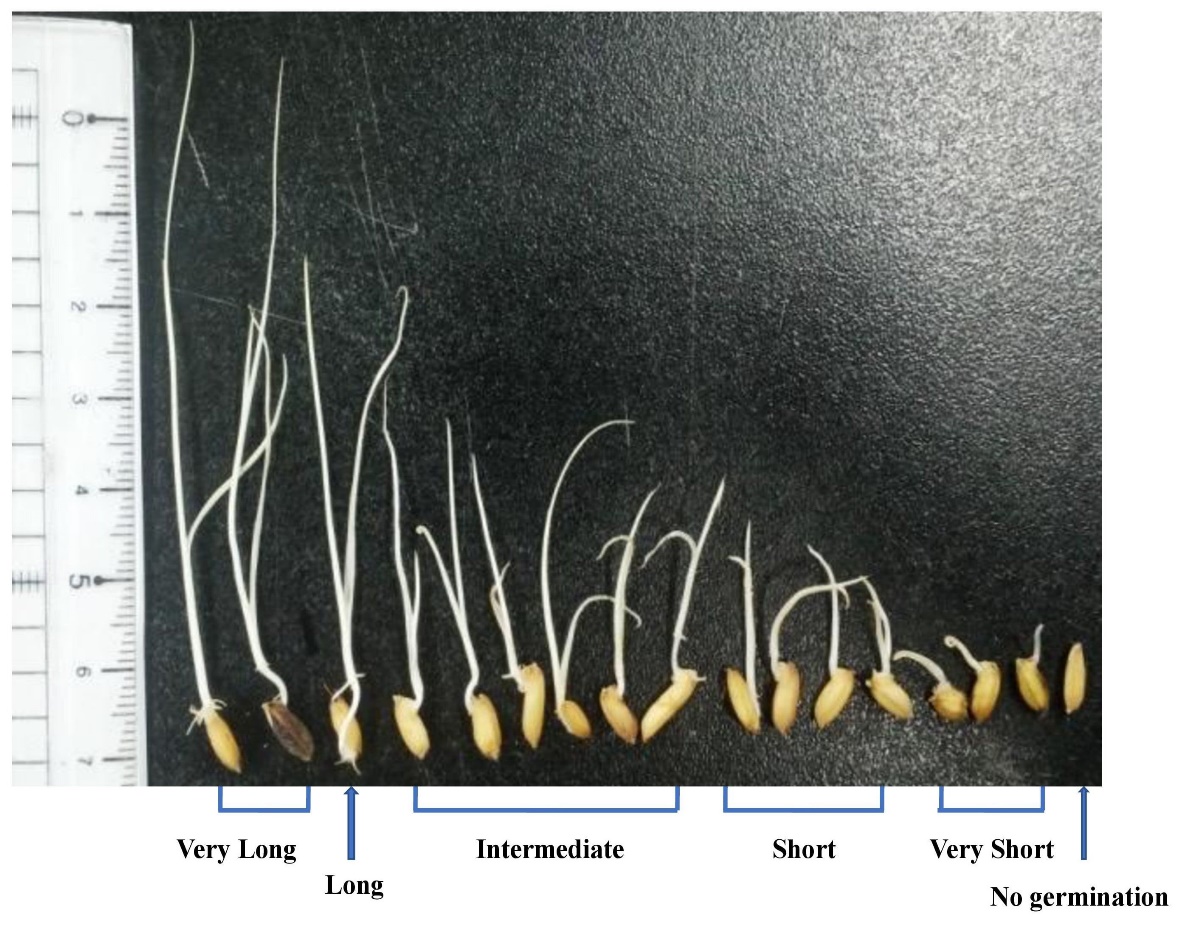


**Supplementary Figure 2.** Phenotypic variation of coleoptile length (in cm) in 421 cultivated rice accessions. The same classification was used for normal coleoptile length (NCL) and flooding coleoptile length (FCL).


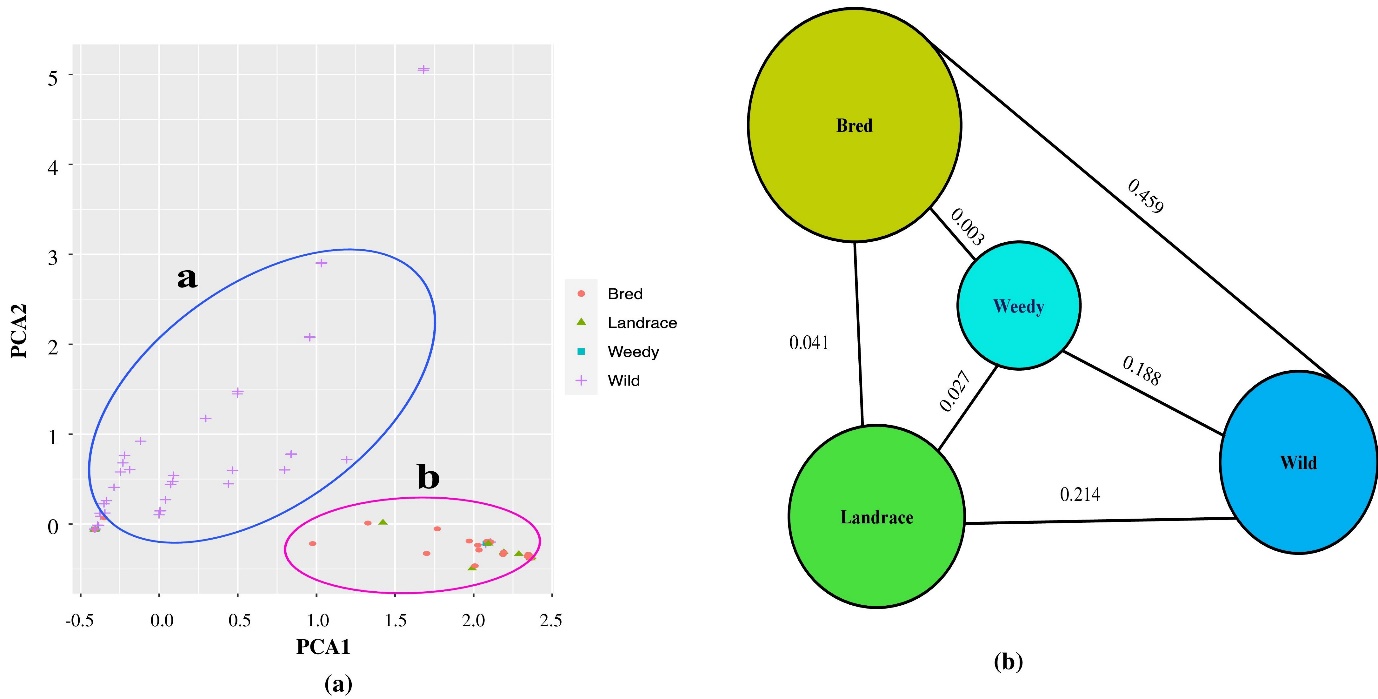


**Supplementary Figure 3.** Principal component analysis (PCA) and genetic differentiation based on the *OsTPP7* gene across varietal types of Korean rice collection. (a) PCA demonstrates the distribution of genetic variables among varietal types. Circle a: distribution of wild accessions, and circle b: distribution of cultivated accessions. (b) *F_ST_* estimates indicate genetic differentiation among the varietal types.


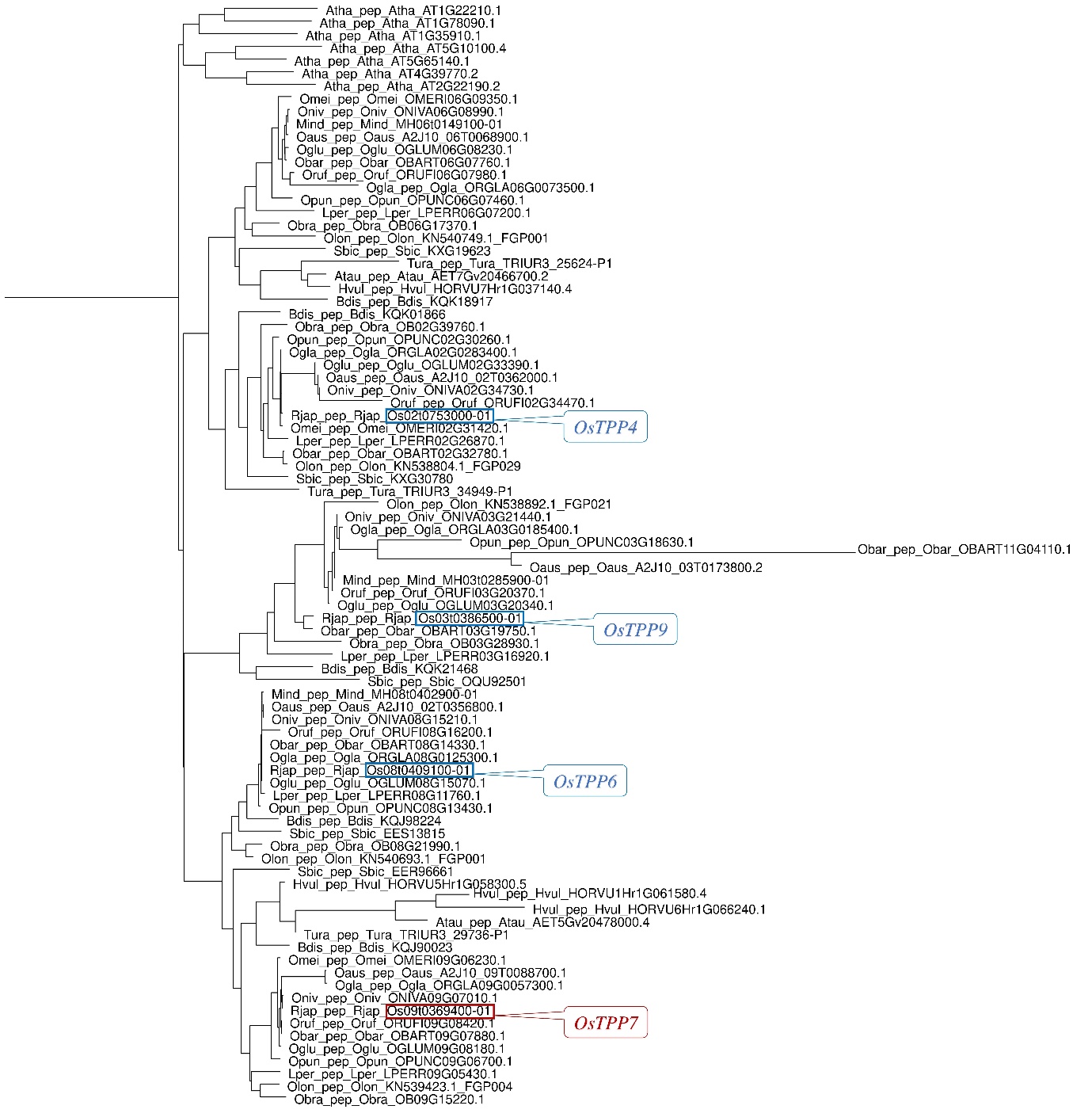


**Supplementary Figure 4:** Phylogenetic analysis of *TPP7* orthologs from 19 plant species.


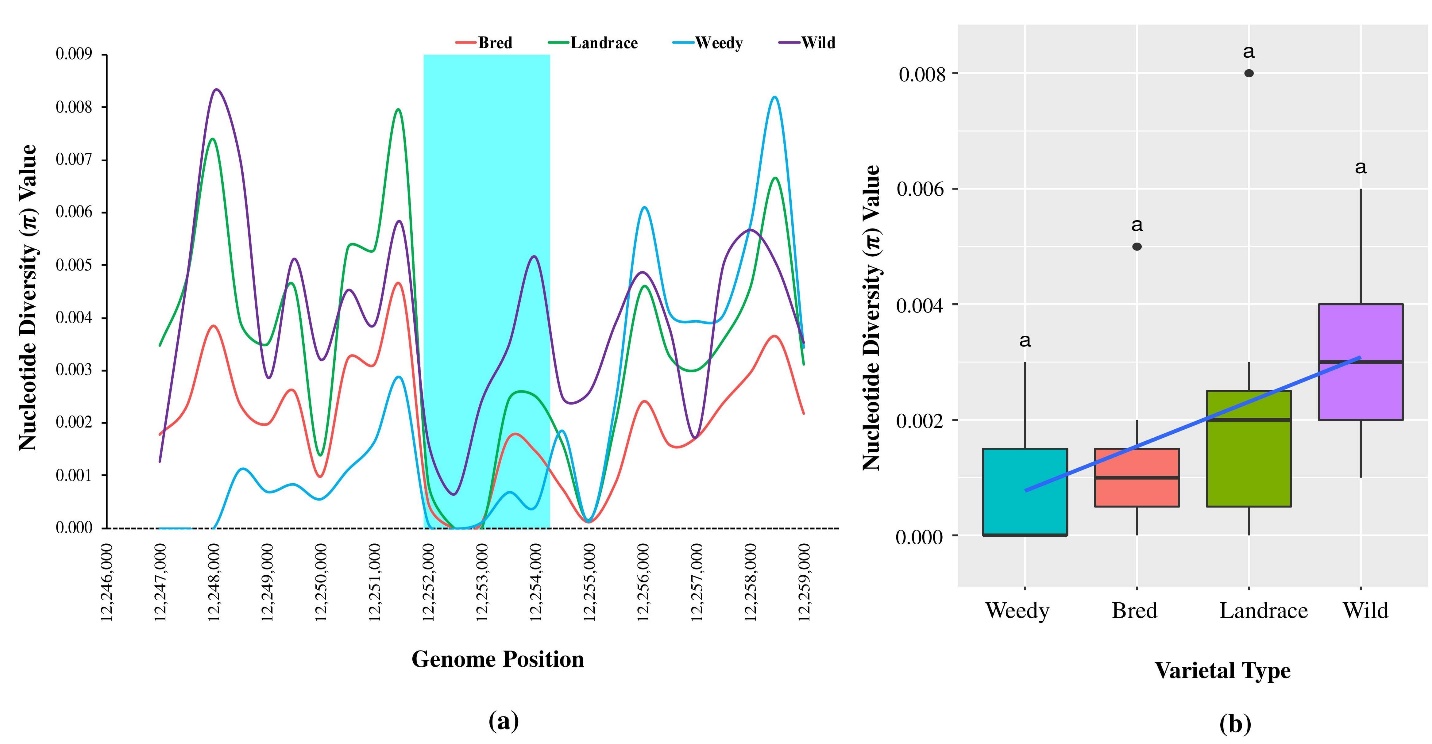


**Supplementary Figure 5.** Nucleotide diversity (π) based on *OsTPP7* across varietal types of the Korean rice collection. (a) Nucleotide diversity values with a 1.0 kb sliding window. The highlighted cyan color shows the *OsTPP7* gene region. (b) Box plots representing a comparison of mean nucleotide diversity among the varietal types. Different letters above each boxplot indicate significant differences among varietal types according to Sheffe's test (p < 0.05).


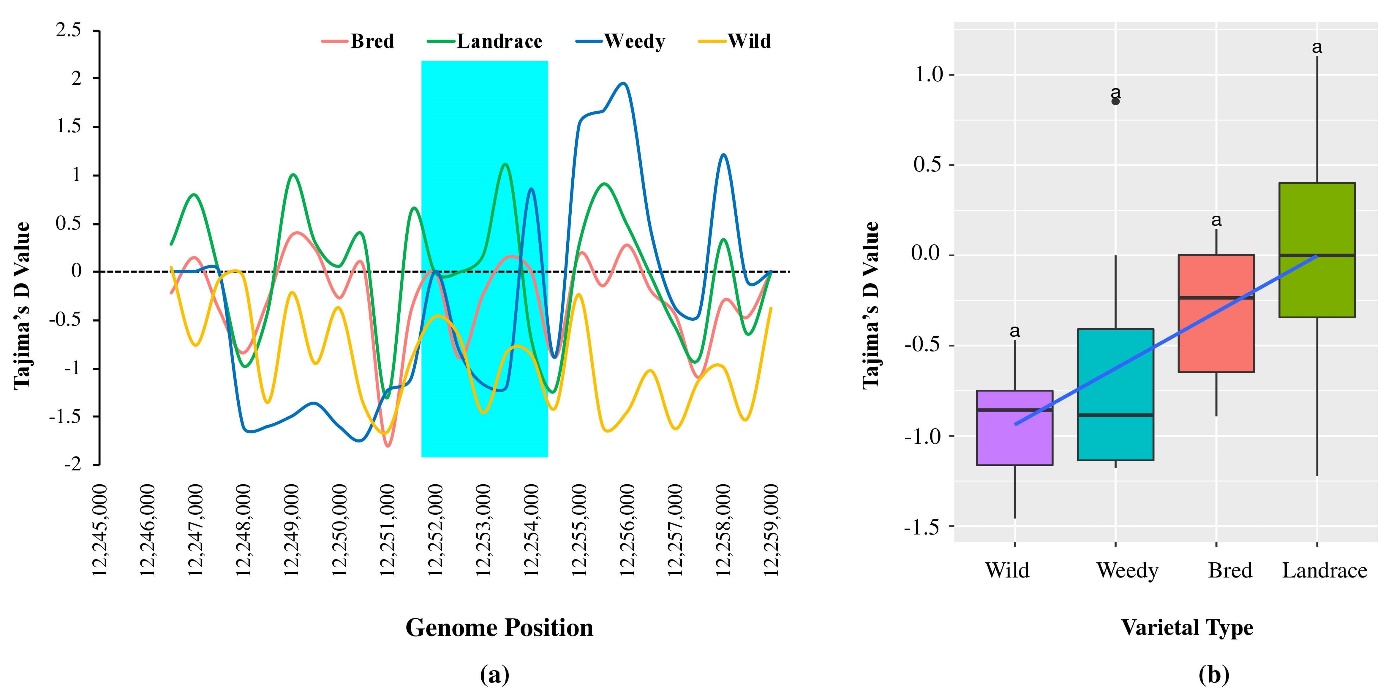


**Supplementary Figure 6.** Tajima's D analysis based on *OsTPP7* across varietal types of the Korean rice collection. (a) Tajima's D values among the ecotypes with a 1.0 kb sliding window and highlighted cyan color indicates the *OsTPP7* gene region. (b) Box plots representing a comparison of mean Tajima’s D values among the varietal types. Different letters above each boxplot indicate significant differences among varietal types according to Sheffe's test (p < 0.05).


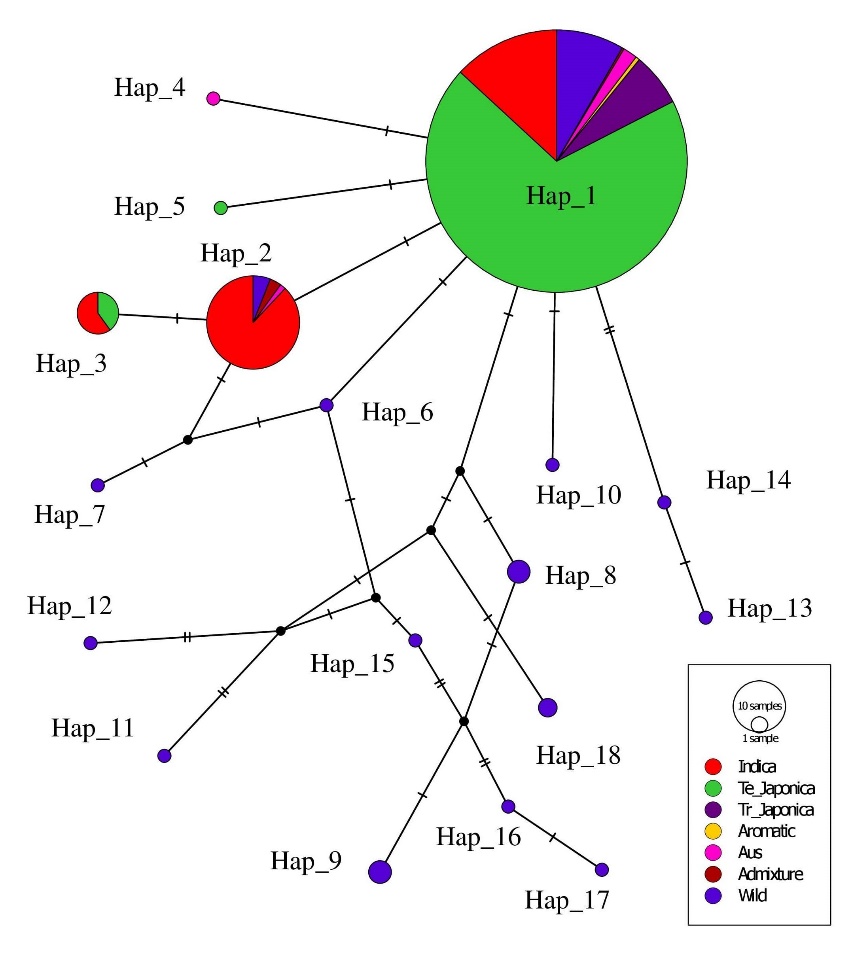


**Supplementary Figure 7.** Haplotype network analysis based on the *OsTPP7* gene from 475 rice accessions. Haplotype network was constructed in the PopART program. The circle size is proportional to the number of samples and ecotypes, and the dashes between haplotypes represent mutational steps between alleles.


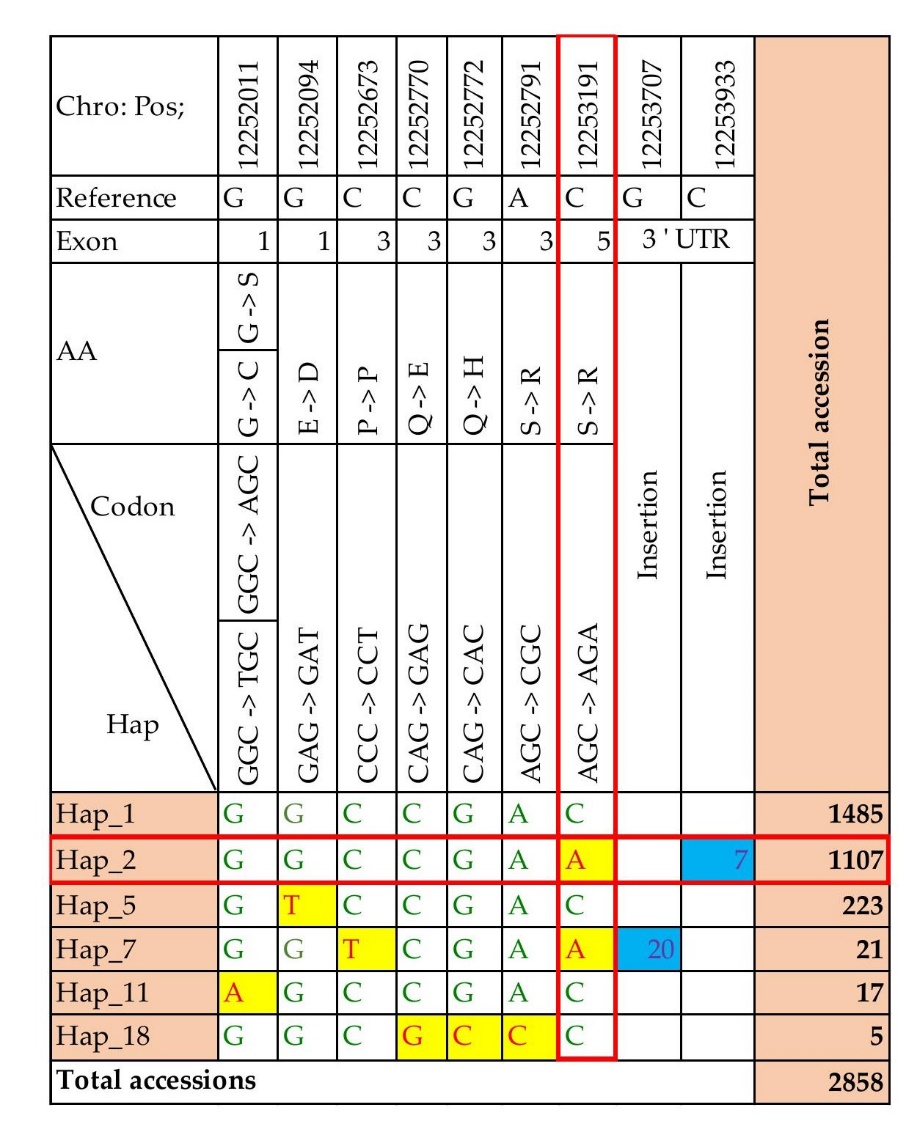


**Supplementary Figure 8:** Details of major *OsTPP7* haplotypes from 3,000 rice accessions (3K_RG) data analysis.

**Supplementary Tables:**

**Supplementary Table 1.** Passport information of the Korean rice collection of 475 accessions, *OsTPP7* haplotypes, and AG phenotyping.

**Supplementary Table 2.** List of the species used for *TPP7* ortholog analysis.

**Supplementary Table 3.** Haplotype analysis of the *OsTPP7* gene from 475 rice accessions. The SNPs are highlighted in yellow, and insertion and deletion are shown with blue and green, respectively. The dash (-) indicate the same as the reference sequence, and "Het" means a heterozygous nucleotide.

**Supplementary Table 4.** List of accessions under each haplotype of *OsTPP7* from 475 rice accessions.

**Supplementary Table 5.** List of *OsTPP7* haplotypes from the analysis on 3000 rice accessions. The SNPs are highlighted in yellow, and insertions and deletions are highlighted in green and blue, respectively. The blanks at the insertion and deletion positions are the same as the reference sequence, and "Het" means a heterozygous nucleotide.

**Supplementary Table 6.** List of accessions under each haplotype of *OsTPP7* from 3K rice accessions.

**Supplementary Table 7.** Phenotyping of selected accessions for AG-related traits in tray-based screening.
